# Integrating general practitioners’ and patients’ perspectives in the development of a digital tool supporting primary care for older patients with multimorbidity: a focus group study

**DOI:** 10.3389/fdgth.2025.1499333

**Published:** 2025-01-21

**Authors:** Ingmar Schäfer, Vivienne Jahns, Valentina Paucke, Dagmar Lühmann, Martin Scherer, Julia Nothacker

**Affiliations:** Department of Primary Medical Care, University Medical Center Hamburg-Eppendorf, Hamburg, Germany

**Keywords:** multimorbidity, chronic diseases, shared decision making, digitalisation, clinical practice guidelines

## Abstract

**Introduction:**

The web application gp-multitool.de is based on the German clinical practice guideline “multimorbidity” and supports mutual prioritisation of treatments by GPs (general practitioners) and patients. The application facilitates sending hyperlinks to standardized assessments by email, which can be completed by patients on any suitable digital device. GPs can document clinical decisions. The tool also supports a structured medication review. Aims of this study were to consider needs and wants of the target groups in implementing the “multimorbidity” clinical practice guideline in a digital tool, and to examine themes of discussions in order to identify which aspects were considered most important for customising a digital tool.

**Materials and methods:**

We conducted six focus groups with 32 GPs and six focus groups with 33 patients. Eight groups were conducted alongside the programming of the web application and four after finishing a prototype. GPs were recruited by mail and asked to invite up to six eligible patients from their practice to participate. Focus groups were based on semi-structured interview guides and discussed assessments, functionalities, usability and reliability of gp-multitool.de. Discussions were transcribed verbatim and analysed using content analysis.

**Results:**

GPs wanted to avoid unnecessary and time-consuming functions and did not want to explore problems that they could not provide solutions for. For some assessments, GPs suggested simplifying scales or including residual categories. GPs and patients also addressed possible misunderstandings due to wording and discussed if some items might be too intimate or overtax patients intellectually. In most cases, participants confirmed usability, but they suggested changes in default settings and pointed out a few minor bugs that needed to be fixed. While some GPs considered data security an important topic, most patients were unconcerned with this issue and open to share their data.

**Conclusion:**

Our study indicates that focus groups can be used to customize a digital tool according to the needs and wants of target groups and thus, improve content, functionality, usability, and reliability of digital tools. However, digital tools still need to be piloted and evaluated in everyday care. In our focus groups, study participants confirmed that gp-multitool.de can be a relevant approach for overcoming deficits in the information needed for mutual prioritisation of treatments by GPs and patients.

## Introduction

Multimorbidity, which is defined as the coexistence of multiple chronic diseases, is a highly prevalent condition affecting more than half of the older primary care patients (1). Many studies indicated that multimorbidity is associated with adverse outcomes such as functional decline, a higher mortality risk and increased healthcare utilisation, e.g., a higher rate of hospital admissions (2, 3). Multimorbidity is challenging for attending general practitioners (GPs), because interactions between diseases and treatments might occur, the benefits of the numerous simultaneous treatments are uncertain and potential harms could result (4). In addition, the management of multimorbidity in primary care demands extra time during consultations (5).

Single condition clinical practice guidelines (CPGs) usually don't consider the complex needs of patients with multimorbidity. They could even lead to an overly high treatment burden for patients, if several CPGs are applied simultaneously (6). After more than twenty-five years of research focusing on multimorbidity, we still have little knowledge about which treatments could improve patient-related outcomes (2). However, mutual prioritisation of treatments between GPs, who usually target the most threatening condition, and patients, who usually aim to reduce the most undesired symptoms, is probably one factor improving the effectiveness of multimorbidity interventions (7).

Generally, there are different options for addressing the complexity resulting from multimorbidity in CPGs (8). A CPG could (a) focus on one condition and take into account specific comorbidities, (b) take into account overall morbidity when focusing on one index condition, (c) focus on a specific combination of conditions, or (d) target multimorbidity itself. One example for the last option is the S3-level clinical practice guideline (CPG) “multimorbidity” (9, 10) by the professional society “Deutsche Gesellschaft für Allgemeinmedizin und Familienmedizin” (DEGAM) in Germany. Backbone of this guideline is the so called “meta-algorithm”, which focuses on mutual prioritisation of treatments and structures decision-making based on the patient's preferences and the medical history shared between GP and patient.

The CPG was piloted with nine GPs and ten patients. In a qualitative interview and focus group study, participants referred to the meta-algorithm as being helpful, but found it to be overly complex. The study participants suggested to develop a digital form of the CPG and advocated for supplementing the meta-algorithm with tools for assessing patient preferences, documenting the social situation and prioritising treatments (11). The strategy for digitalisation of the DEGAM emphasizes that involving users and piloting under real world conditions are important parameters in the development of digital solutions (12).

Following these suggestions, we developed gp-multitool.de, a digital tool supporting GPs in the evidence-based treatment of patients with multimorbidity. The tool was based on recommendations from the DEGAM CPG “multimorbidity”, and their specific implementation should consider needs and wants of GPs and their patients with multimorbidity. Therefore, we analysed the perspective of GPs and patients towards gp-multitool.de in order to improve the tool. Aim of this study was to examine themes of these discussions in order to identify which aspects are most important for GPs and patients in customising a digital tool.

### Concept for gp-multitool.de

In order to support the evidence-based treatment of patients with multimorbidity, recommendations of the 2017 DEGAM CPG “multimorbidity” (9) were implemented in gp-multitool.de. Main functionalities comprised standardised assessments, which should aide GPs’ information management. Additionally, medication reviews and talks between GP and patient about assessment results were supported by the tool. Recommendations of the DEGAM CPG “multimorbidity” and their implementation in gp-multitool.de are shown in Table 1.

**Table 1:** Implementation of recommendations from the DEGAM CPG “multimorbidity” (2017).

Recommendation in DEGAM CPG “multimorbidity”	Implementation in gp-multitool.de
When determining patient preferences and values, the following aspects should be addressed: • Patients should be encouraged to express their personal goals and priorities. This includes clarifying the importance of: (a) Maintaining social roles in occupation/work, participation in social activities, family life; (b) Preventing specific events (e.g., stroke); (c) Minimising side effects of medication; (d) Reducing the burden of treatment; (e) Prolonging life. • Patients’ attitudes towards their therapy and its potential benefits are to be explored. • It should be clarified with the patient whether and to what extent partners, relatives or caregivers should be involved in important care decisions	Standardised assessments of • Prioritisation of treatment goals, • Control preferences, • Activities and participation, • Social contacts, • Pain, • Anxiety and depression, • Other health complaints, • Treatment burden, and • Preferences for involvement of partners, relatives and caregivers.
Continuous comparison of patient's and physician's priorities is the essential prerequisite for good decisions. Every decision should be made against the background of patient preferences, which often only develop during the discussion, and the joint prioritization of treatment goals. This can refer to both increasing and decreasing the intensity of treatment. In this case, a comparison should be made between the physician's priorities (e.g., prevention of avoidably dangerous courses of disease) and the patient's priorities (e.g., fear of loss of autonomy).	• Reminder for discussions between physician and patient regarding the results of standardised assessments and “brown bag” review of medication. • Documentation of the results of these discussions.
• It should be determined whether other medical or non-medical healthcare professionals have been consulted since the last consultation and with what result. • If multiple health professionals are involved in the treatment of patients with multimorbidity (patient, specialists, family doctor, relatives, nursing staff), they should coordinate diagnosis and therapy.	Assessment of involvement of other healthcare professionals.
During drug treatment, the medication actually used should be evaluated. At the same time, misunderstandings about indication, effect and method of intake or application should be clarified and eliminated.	• “Brown bag” medication review. • Standardised assessment of problems with medication.

CPG, clinical practice guideline; DEGAM, Deutsche Gesellschaft für Allgemeinmedizin und Familienmedizin (“German Society of General Practice and Family Medicine”).

The implemented assessments were standardised, ie, each of them consisted of a predefined set of questions mostly with predefined answer categories. Assessments comprised two validated instruments for the assessment of social contacts (14) and psychiatric symptoms (15). Additionally, based on the literature (16–21), seven instruments were developed by the study team. They comprised prioritisation of treatment goals, assessments of control preferences, involvement of other healthcare professionals, activities and participation, treatment burden, problems with medication, pain, and other health complaints. The final versions of these instruments are shown in Boxes S1 through S7 in Supplementary Material.

We designed gp-multitool.de as web application for GPs and their practice teams. It facilitates sending hyperlinks to the standardised assessments by email. The assessments then can be completed by patients on any mobile or stationary digital device with browser and internet access. Screenshots of the questionnaires on mobile devices are shown in Figure 2. It is also possible to conduct and document assessments during consultations. Moreover, assessment forms can be printed by the GP, filled out by patients using pencil and paper and afterwards entered manually in the digital tool by the practice staff. GPs need to approve finished assessments, talk with patients about the results and they can document their mutual decisions in gp-multitool.de. A screenshot of these functions is shown in Figure 3. Additionally, the GP can access a history with older assessments and decisions.

**Figure 1:**
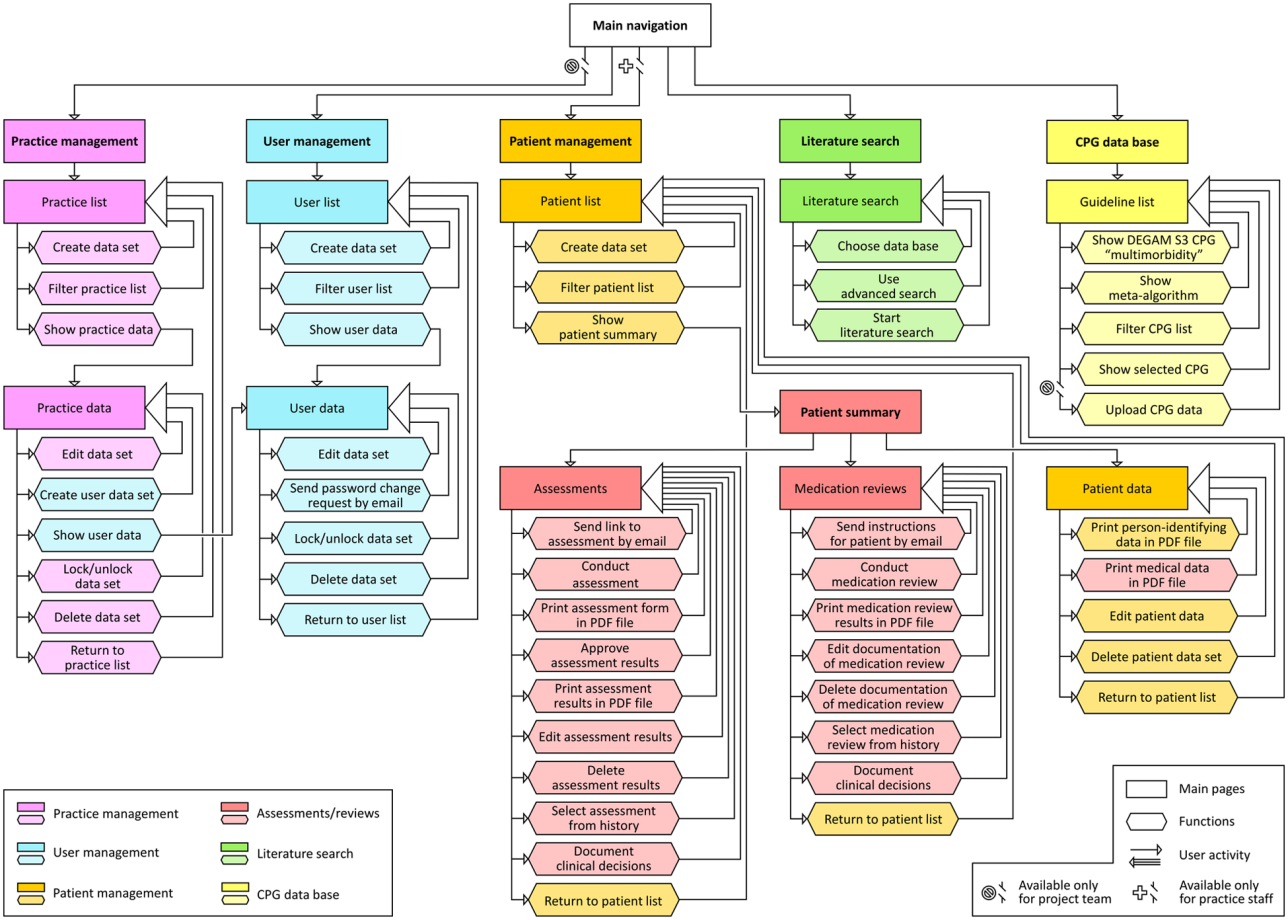
Sitemap of gp-multitool.de. CPG, clinical practice guideline; DEGAM, Deutsche Gesellschaft für Allgemeinmedizin und Familienmedizin; PDF, adobe portable document format.

**Figure 2:**
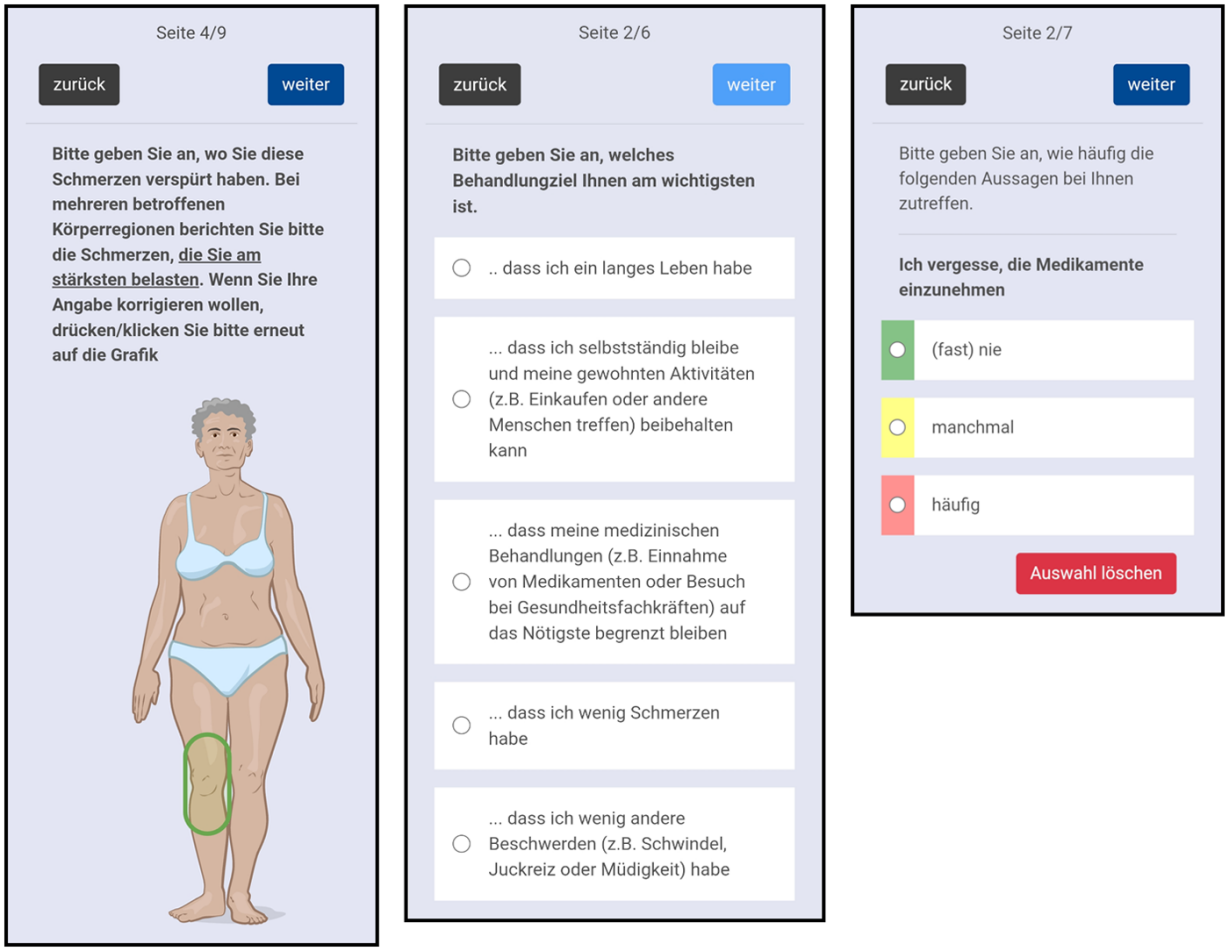
Screenshots of patient assessments on mobile devices. Image © Janis Vernier 2022.

**Figure 3:**
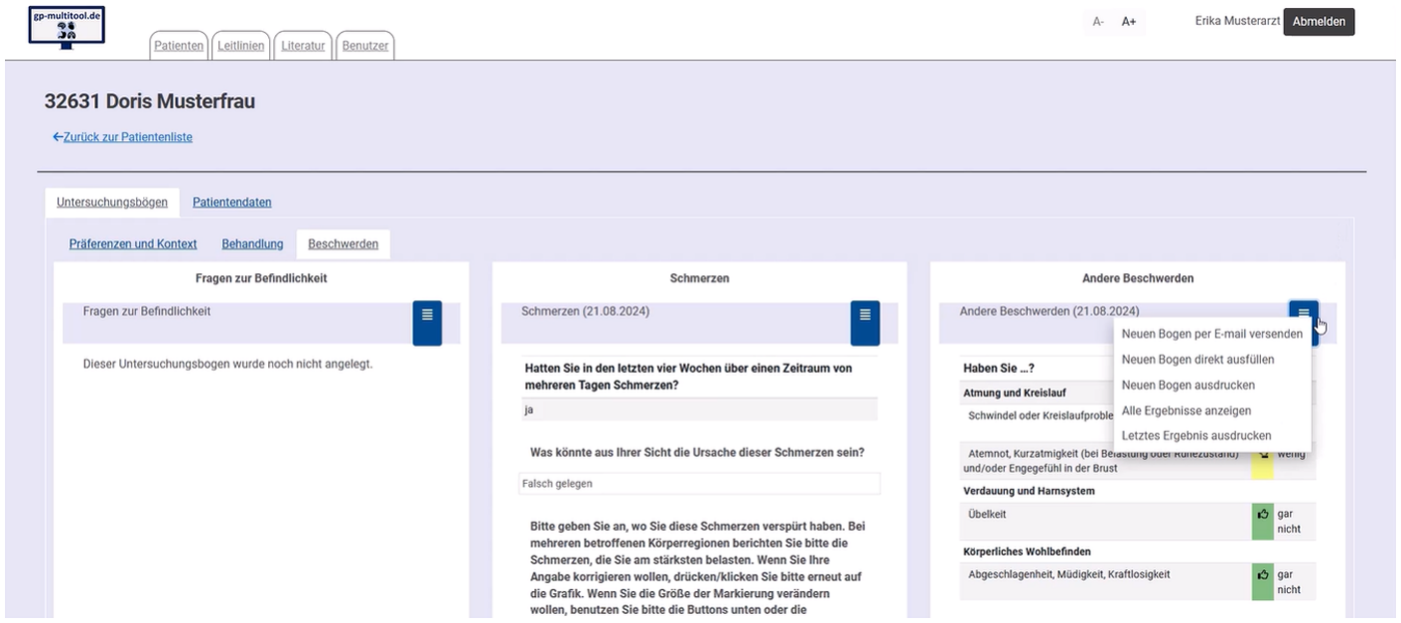
Screenshot of GPs’ management of assessments.

The concept for gp-multitool.de also comprised other functions including easy access to structured literature search and a database of clinical practice guidelines relevant for primary care, which can be updated within gp-multitool.de by the study team. Moreover, systems for managing practices using gp-multitool.de, users within the practices and patients under treatment were implemented. A site map of gp-multitool.de including all available functions can be found in Figure 1.

Measures of data protection and data security were defined in a data protection concept including data protection impact assessment. We based this concept on the Standard Data Protection Model of the Conference of the Independent Data Protection Authorities of the Federal and State Governments in Germany (22) and the Technical Guideline for Security Requirements for eHealth Applications of the German Federal Office for Information Security (23). Additionally, the company NSF Prosystem GmbH, Hamburg, was consulted regarding requirements resulting from national and European law regulating medical devices. The application gp-multitool.de was programmed by Trinidat Software-Entwicklungs-GmbH, Düsseldorf, who also provide technical support.

## Materials and methods

We conducted a qualitative study based on twelve focus groups with GPs and their patients. The first eight focus groups were conducted in parallel with the programming of gp-multitool.de. After the programming of a prototype was finished, gp-multitool.de was tested and discussed in four additional focus groups. The study was approved by the Ethics Committee of the Medical Association of Hamburg on 27 June and 5 September 2022 (file number 2022-100786-BO-ff) and reported according to the COREQ checklist (13).

### Project advisory board

In order to facilitate patient and public involvement, a project advisory board was recruited, which consists of representatives of the patient organisation “Bundesarbeitsgemeinschaft Selbsthilfe e.V.”, the caregiver organisation “Wir pflegen e.V.”, the physician organisation “Deutscher Hausärzteverband Landesverband Niedersachsen e.V.”, the professional society DEGAM, the Schleswig-Holstein Association of Statutory Health Insurance Physicians, the health insurance company “Techniker Krankenkasse” and the private company “GAIA AG”. The board advised the research group on how to establish interests and concerns of patients and care providers in the design, implementation and evaluation of gp-multitool.de. For example, it commented on the recruitment process for the focus groups, assessed data protection and discussed functionality and usability of gp-multitool.de before the focus groups were conducted.

### Researchers

The focus groups were conducted and analysed by three researchers. JN is a female health educator working as junior researcher at university. VJ is a female physician working in a regional hospital. IS is a male sociologist, doctor of philosophy and private lecturer for epidemiology and health care research working as senior researcher at university. JN and VJ were trained in qualitative research and supervised by IS. No researcher had any relationship with any study participant and, before participation, study participants had no relevant knowledge about the researchers.

### Participants

Study participants were recruited in two steps. In the first step, 663 GPs in the region of Hamburg, Germany, and rural surroundings were identified using the websites of the regional Associations of Statutory Health Insurance Physicians and invited by mail to participate in the study. GPs were eligible if they worked as statutory health insurance physician. In case of interest, GPs declared their consent in a contract with the study centre.

The second step consisted in selecting patients by applying convenience sampling. Participating GPs were asked to identify up to six eligible patients from their practice and to invite them during their regular consultations to participate in the study. Patients were included, if they were 65 years or older and suffered from at least three chronic diseases. Patients were excluded if they had no capacity to consent (e.g., in case of dementia), if they were not able to participate because of medical reasons (e.g., severe psychiatric disease), if they were not able to hear, if they had insufficient German language skills or if they lived in a nursing home. Patients received study information by their GP and had to sign an informed consent sheet before participation.

Each focus group with GPs had between 4 and 6 participants and 5.3 ± 0.8 participants on average. In total, 32 GPs participated. Of those, three (9.4%) were 40 years or younger, four (12.5%) were between 41 and 50 years old, 22 (68.7%) were between 51 and 60 years old and three (9.4%) were older than 60 years. Most GPs (25; 78.1%) were women and 7 (21.9%) were men. Mostly experienced GPs were included into the sample. Only five GPs (15.6%) had been working for ten years or less in primary care, while six (18.8%) had been working between eleven and 20 years, 17 (53.1%) between 21 and 30 years, and four (12.5%) since more than 30 years.

Each focus group with patients had between 3 and 8 participants and 5.5 ± 2.1 participants on average. Of the 33 patients who participated, ten (30.3%) were between 65 and 70 years old, 19 (57.6%) between 71 and 80 years old, and four (12.1%) were 81 years or older. The fraction of women (19; 57.6%) in the sample was a little higher than the fraction of men (14; 42.4%). Educational level of twelve patients (36.4%) was primary, while 19 (57.6%) attained secondary education and two (6.1%) tertiary.

### Focus groups

Four GP groups and four patient groups were used to discuss benefit, wording and risk of bias of the standardised assessments, before they were implemented in gp-multitool.de. The interviews were based on pre-defined semi-structured interview guides (24), which reflected our research aims and the CPG recommendations. Different interview guides were used for focus groups with GPs and patients (25, 26). Discussed stimuli in GP focus groups included:

• Which information is beneficial for making treatment decisions with older patients with multimorbidity and which information is unnecessary or even harmful? Why?
• Please think of older patients with multimorbidity that you know from your practice. Do you think they understand the questions and would they be able to give adequate answers? Which problems might occur and how could they be avoided?
• Would you assume, that there is a risk of bias, e.g., due to social desirability? What is your experience in talking about these topics? Do you have any suggestions for framing or alternative wording?

Discussed stimuli in patient focus groups included:

• How do you feel about your GP collecting information about you in this way?
• Do you find the questions understandable? Why?
• How would you feel about talking to your doctor about these issues?

The current versions of the assessments were shown during discussion. After each focus group, the seven self-developed assessments were modified considering the feedback of the study participants. The two validated instruments were discussed to evaluate if they were suitable for the target group, understandable and beneficial for treatment decisions, but not modified.

After programming of the prototype was finished, gp-multitool.de was tested and discussed in the remaining two GP groups and two patient groups. In the GP groups, all relevant functions with focus on patient management, management of assessments and medication reviews, data collection, presentation of results, and documentation of treatment decisions were tested and discussed afterwards. Stimuli in GP focus groups included:

• Which problems do you expect when using gp-multitool.de? Which improvements would you like to see? Why?
• What are your thoughts on the menu navigation? Is it intuitive and easy to use without further instructions?
• Would you use the tool in this form in your practice? Why?

During the patient focus groups, data collection was tested on the patients’ own mobile devices and discussed afterwards. Discussion of assessments was also possible. Discussed stimuli included:

• Were the provided texts helpful and understandable? Were all the buttons understandable and easy to find? Which questions came up? Which problems occurred? Why?
• Were you able to operate the program? Which improvements would you like to see? Could you use the program on your own or would you need help from other people? Why?
• How do you like the design of the application? How do you think about specific design elements, e.g., the presentation of answer categories, colour schemes, and font sizes?

### Data collection

Study participants were contacted by email or telephone in order to coordinate dates for focus groups. Of the GP groups, one was conducted in person at the research institute and five were conducted online via WebEx. Of the patient focus groups, three were conducted in person at the research institute, two in person at a community centre in a rural area and one was conducted online via WebEx. Each focus group lasted approximately two hours. Most focus groups were moderated by two researchers (IS, JN) and in the other a third researcher (VJ) was involved. During the focus groups, a student assistant took field notes. All focus group discussions were audio recorded and transcribed verbatim.

### Analysis

Transcripts were analysed using structuring content analysis (27) based on deductive and inductive coding. In a first step, categories were extracted from the interview guide. During coding, the category system was refined using the analysed data and relevant literature (28). Two researchers (JN, VJ) independently coded the data and developed the first category system. Codes and examples were discussed between JN, VJ, and IS until consensus was reached. Based on this consensus, the final category system was developed. MAXQDA Analytics Pro 2022 (version 22.3.0) was used to support data analysis.

## Results

During the discussion of gp-multitool.de, ten themes emerged: acceptability, adequacy, correctness, relevance, reliability, security, suitability, understandability, usability, and validity. Operationalisation of the themes is shown in Table 2. The themes of the discussion of the different assessments and functions of gp-multitool.de are shown in Table 3. In Supplementary Table S1A through S1d in Supplementary Material, exemplary quotes of study participants are given.

**Table 2:** Operationalisation of themes in focus group discussions.

Theme	Operationalisation
Acceptability	Target group is willing/unwilling to use the function in the intended manner
Adequacy	Contents probably cause/do not cause undesirable side effects, e.g., discriminating against patient groups, giving patients false hope
Correctness	Contents are verified/falsified by current medical evidence
Relevance	Contents are beneficial/not beneficial for making treatment decisions with older patients with multimorbidity
Reliability	Functions operate well/less well
Security	Data protection and data security are well/less well implemented
Suitability	Contents are well/less well tailored to the target group
Understandability	Patients probably can/cannot interpret the content as intended
Usability	Functions can be used more/less conveniently
Validity	Contents represent a construct more/less accurately

**Table 3:** Themes by assessments and functions of gp-multitool.de.

	Acceptability	Adequacy	Correctness	Relevance	Reliability	Security	Suitability	Understandability	Usability	Validity
Prioritisation of treatment goals	X	–	–	X	–	–	X	X	–	X
Control preferences and involvement of other healthcare professionals	X	X	–	X	–	–	X	X	–	X
Social contacts	–	X	–	X	–	–	–	–	–	X
Activities and participation	X	–	–	X	–	–	X	–	–	X
Problems with medication	X	–	–	X	–	–	X	X	–	X
Treatment burden	–	–	–	X	–	–	X	–	–	X
Pain	–	X	–	–	–	–	X	X	–	X
Anxiety and depression	–	–	–	X	–	–	–	–	–	–
Other health complaints	–	–	X	X	–	–	X	X	–	X
Medication reviews	X	X	–	X	–	–	X	–	X	–
Management of assessments, patients and users	–	–	–	X	X	X	X	X	X	X
Literature search and CPG data base	–	–	–	X	–	–	–	–	X	–

X, the respective topic was discussed; –, the respective topic was not discussed.

### Assessments

#### Prioritisation of treatment goals

Prioritisation of treatment goals was one of the most discussed instruments. While most GPs found the assessment relevant, some also were concerned about acceptability. One patient emphasized that prioritization of treatment goals was not easy. There has been an extensive revision of this instrument based on feedback of the study participants in multiple focus groups. Several GPs recommended to change the wording, for example regarding the alternatives “to prolong life” vs. “to have a long life”, “to combat pain” vs. “to have little pain”, and “autonomy” vs. “independence”. Initially, treatment goals were rated on a percentage scale. In order to increase validity and suitability of the assessment, some GPs and patients suggested simply to rank the items according to their priority.

#### Control preferences and involvement of other healthcare professionals

Some GPs underlined the relevance of knowing the degree of control the patients prefer for their treatment and whether other people such as relatives or caregivers should be involved in treatment decisions. Other GPs emphasized that the assessment of control preferences could raise expectations they could not agree with. Some GPs questioned whether the assessment of control preferences was understandable, whether the wording was valid and whether it was suitable for the target group, e.g., whether patients could be overtaxed intellectually. Following this discussion, the first version of this assessment was replaced by a new version that was more in line with the GPs’ suggestions. Patients discussed understandability and adequacy of the assessment. For example, the first response option contained being “overwhelmed by having to make a decision”. One patient found this option not acceptable, while another patient underlined the validity of this option.

#### Social contacts

Initially, a summary score had been calculated from the assessment of social contacts. GPs and patients found the selected assessment relevant, but some GPs questioned the validity of the summary score. Instead, they suggested to use the existing item “satisfaction with the social situation” as alternative summary measure. They also discussed the adequacy of the introductory text of the instrument and suggested a more neutral wording. Items and response categories of the instrument were widely accepted among study participants and remained unchanged.

#### Activities and participation

Several GPs emphasized the relevance of the items, but criticised that the large range of functional levels included in the instrument which might impair the acceptability of the assessment. Some GPs suggested to change items and response categories in order to increase suitability, validity and acceptability of the assessment. For example, they recommend to shorten the sentences and proposed that “looking after the belongings” and “managing money” should be removed from the assessment. Moreover, they discussed which aspects should be included regarding “eating”, “keeping oneself mentally fit”, “physical activities”, and “social activities”. Patients discussed the relevance and validity of items and response categories. For example, they suggested to add the residual category “not applicable/not important for me” for every item, to include grandchildren and pets as examples for “those who depend on them”, and to focus more on sports and exercises regarding “physical activities”. Following the discussion, we removed seven of the original 15 items, added two, and completely revised another two items.

#### Problems with medication

GPs regarded the instrument as relevant, but suggested to shorten it and to change the introductions, specific items, and response categories in order to increase validity, suitability and acceptability of the assessment. Patients found the instrument relevant and understandable, discussed validity of items and response categories and suggested to differentiate between the different medications they use. Based on participants’ feedback, we reduced the number of response categories and removed three of the original five items, added one and split another item into two in order to allow for more differentiated answers.

#### Treatment burden

There was a controversial discussion among GPs regarding the relevance of the instrument and its specific items, e.g., “paying for the medication yourself”, “keeping agreed appointments”, “making lifestyle changes”, “being dependent on family and friends”, and “consultations in GP practices”. For example, some GPs emphasised that problems like transport could not be solved by GPs and they therefore did not want to raise false hopes by talking about these items.

GPs suggested to increase validity and suitability of the assessment by partially changing the wording and using only few response categories. They also commented on the scale, which specific response categories should be used, and they also found the specific items more helpful than a possible summary score. Following the discussions, we removed seven of the original 13 items, added three new items and completely revised another three items. We also reduced the number of response categories.

#### Pain

In order to increase validity of the instrument, GPs commented on items and response categories. For example, they debated if it was better to measure the location of every pain or only the pain with the highest intensity and if current pain intensity or average pain intensity should be measured. They also discussed the categories for the quality of pain and their wording, e.g., stabbing, dull or burning. GPs also commented on the adequacy and suitability of the illustrations used for localizing pain. For example, they discussed how to avoid discriminating against other cultures, how to represent different genders, and if the pictures accurately represented usual patients in the age group 65 years and older.

Patients found the assessment understandable and the illustrations helpful, but discussed the adequacy of the colour scheme used in the visual analogue scales. Most patients appreciated it or found it helpful, while one patient emphasized his perception of red as a signal colour. As presumed by GPs, some patients had problems to describe their pain qualities. One patient suggested to include subjective reasons for pain into the assessment. Based on the participants’ feedback, we changed the illustrations, removed two items, added another two, and completely revised three items.

#### Anxiety and depression

This instrument was well known by several study participants and some GPs already used it regularly in their consultation in pencil and paper form. These study participants reported positive experiences with its use in their elderly patient population. For these reasons, the instrument was implemented in gp-multitool.de without any changes.

#### Other health complaints

The patients found the instrument suitable and understandable, and several patients underlined the relevance of the instrument. The instrument also was seen as relevant and suitable by most GPs. However, GPs also gave many suggestions how to increase correctness and validity of the assessment. For example, they suggested to combine twelve specific items into five larger categories, divided one other specific item into two smaller categories, and seven items were seen as not relevant while two items were missed by some GPs and therefore added to the instrument. They also had suggestions how to refine the wording, the order of the items and the assignment of items to organ systems.

### Functions

#### Medication reviews

GPs found the implemented invitation letter helpful and emphasised the importance of including over-the-counter drugs into the review, but had concerns regarding the duration of the medication reviews. Therefore, the respective wording was changed, but implemented procedures remained as intended.

#### Management of assessments, patients and users

After testing the tool, GPs emphasized the usability of patient and user data base and underlined that the tool was helpful by providing different digital assessments. GPs suggested to reduce the complexity of the tool and save user time by removing unnecessary functions from the GPs’ perspective such as a dash boards indicating new events. They also suggested to facilitate data exchange with their practice management systems, but this function could not be realised due to technical difficulties.

GPs found the presentation of assessment results useful, appreciated the colour scheme of the response categories and suggested to use only short text without examples when presenting results. They also discussed the relevance of reminders for patients to fill out digital assessments, the validity of assessment results over time and had suggestions for changing the wording in some functions. Generally, digital assessments were seen as good approach, but there was a controversial discussion if digital assessments were suitable for their older patients with multimorbidity. Some GPs suggested in this context that relatives could give support if patients faced problems in using the tool.

During the usability test of the assessments, patients identified that gp-multitool.de did not work the same way on different mobile devices, which had to be fixed. Generally, they found the tool convenient to use, but also had some suggestions on how to improve the usability of the assessments, e.g., by not using pre-filled radio buttons as default setting in the patient assessments. In the discussion, some GPs acknowledged the importance of data protection, but several patients stated that they were not concerned regarding this topic.

#### Literature search and CPG data base

GPs found the CPG data base and literature search usable, but there was a controversial discussion about the relevance of these functions. Some GPs found both helpful, but others stated that they would use other sources for finding literature and CPGs. The discussions did not result in any substantive changes in these functions.

## Discussion

### Statement of principal findings

For both GPs and patients, the relevance of assessments and functions was an intensively discussed theme. GPs did not want to raise false hopes by exploring topics they could not offer to help with. They also wanted to avoid unnecessary and time-consuming functions. Validity and suitability of the assessments were frequently discussed, but mostly by GPs. Sometimes, they suggested simplifying scales and response categories or including residual categories. Patients supported such changes.

Understandability was relevant for both, GPs and patients, and also a frequently discussed theme. In particular, they addressed possible misunderstandings due to the wording of items. Adequacy and acceptability were a thematic focus in less than half of the assessments. GPs and patients discussed if some items might be too intimate or even discriminatory or if they might overtax patients intellectually. Correctness of the items was intensively discussed, but mostly regarding the clustering of health complaints in one questionnaire.

GPs and patients assessed and in most cases confirmed usability of all functions, but patients suggested changes in default settings and GPs recommended to facilitate data exchange with GPs’ practice management systems. Reliability was an important aspect in the discussion of the management of assessments, patients and users, where study participants pointed to a few minor bugs that needed to be fixed. There was little discussion regarding data security. While some GPs considered it an important topic, most patients were unconcerned with this issue and open to share their data.

### Strengths and limitations

The application “gp-multitool.de” was conceptualised as digital support for the implementation of the DEGAM CPG “multimorbidity”. As such, the concept was evidence-based. Patients and public have been involved via the project advisory board. And needs and wants of the target groups have been integrated by considering the feedback given in the focus groups described here.

In the focus groups, both, the healthcare providers’ and consumers’ perspectives have been taken into account which facilitates exploring complementary or even contradictory views (29). Also, patients and GPs of different sexes, age groups, experience and educational levels are represented in our sample. Moreover, intersubjective comprehensibility and credibility of the results have been enhanced by two researchers independently coding the material and consensus regarding the category system between three researchers.

Limitations include low response rate of GPs and self-selection of participants, which could result in higher probability for participation of individuals with certain characteristics (30, 31). For example, patients with higher symptom burden and GPs with higher affinity for digitalisation could have been more motivated to participate. Still, some participating GPs were sceptical or even rejected the idea of digital exchange of information between GPs and patients. Another group that may be underrepresented in our study are patients who do not have the necessary resources and skills to handle digital assessments. Therefore, sometimes the discussion of suitability did not include the concerned persons.

In order to receive advice of the target groups regarding how to improve gp-multitool.de, we discussed the quality of the tool in semi-structured focus group discussions. With this approach, we neglected standardised methods for quality assessment of technology like UTAUT (“unified theory of acceptance and use of technology”) (32) and possibly missed specific details on the acceptance of gp-multitool.de.

In our study, GPs and patients discussed separately, i.e., patients could not reply to comments of GPs and vice versa. Therefore, misunderstanding of patients about the perspective of GPs and misunderstandings of GPs about the perspective of patients could not be identified in our study. Also, it is possible that views on data protection and other aspects of the tool are varying in other regions of the world due to different cultural identities and healthcare systems.

### Comparison with the literature

Focus group discussions are often used for developing new scales (33–35), or to assess face and content validity of existing scales (36–38). Also, qualitative studies can support the development of digital tools. For example, in a study from Norway, interviews, focus groups and a workshop were used to accompany content and system development of a digital tool supporting shared decision making in the health care for patients with chronic conditions (39). In our study, a similar approach was used by refining content, functionality, usability and reliability of gp-multitool.de via focus group discussions. In line with other studies (40, 41), our results showed that comprehensibility and acceptability needed to be improved.

In addition to the wording and content of the questionnaires, the participants also discussed the relevance of the surveyed topics. Similar to a Swedish focus group study on attitudes towards digital questionnaires to prepare medical appointments for patients with diabetes (42), both, physicians and patients welcomed that gp-multitool.de supports a more active participation of patients in medical decision making. In contrast to the cited paper, the specific wording of items was of high relevance to the physicians in our study. For example, they wanted to avoid misunderstandings or to raise false expectations in their patients with multimorbidity, which punctually led to extensive rewording and revising of the assessments.

One factor that was particularly discussed by the participating GPs was the risk of revealing problems that, on the one hand, cannot be ignored, but, on the other hand, could not be addressed, e.g., due to time constraints. In another interview study (43) using a digital tool to assess patient-reported outcomes prior to healthcare visits, many issues were uncovered by healthcare professionals, but they had little time to resolve these issues, which was experienced as challenging by study participants. In gp-multitool.de, we revised content and functions, which lead to undesirable side effects from the perspective of the target groups.

In our study, there was a large difference between GPs and patients regarding the subjective importance of data security. While GPs generally stressed the relevance of security aspects, many patients did not see data security as a priority. Other studies on this subject show conflicting results. For example, in a German mixed methods study on privacy and data protection in eHealth, older patients with worse health condition had less concerns about privacy and data protection than younger and healthier patients (44). Another mixed methods study about privacy concerns of older adults using voice assistant systems came to similar conclusions (45). In contrast, several other studies stressed a higher awareness and more cautious behaviour among older people (46–48).

### Implications

In many countries including Germany, policy makers advocate for speeding up processes of digitalisation, particularly in the medical care system (49–52). This requires the development of manifold digital solutions. Digital tools can help to reduce the high level of complexity, which often applies to the care of older patients, in particular, in case of multimorbidity (53–55). Our study indicated that focus groups can be used to customize digital tools according to the needs and wants of the target groups and thus, improve content, functionality, usability, and reliability of such tools. However, this is only a first step in participatory development. The next steps are to pilot and evaluate the digital tools in everyday care to assess if they are feasible for implementing in healthcare, and effective in improving patient-relevant outcomes (60, 61).

Central aim of gp-multitool.de was evidence-based digital support for mutual prioritisation of treatments between GPs and patients. Many studies indicated deficits in the knowledge needed for this process. For example, a study from the Netherlands (56) showed low agreement in prioritisation of treatment goals of patients and the perception of both their GPs and medical specialists. Similar results were found for other topics assessed in gp-multitool.de. For example, GPs had little knowledge on patients’ social relations and feelings of loneliness (57). And information management regarding medication adherence needed to be improved, both regarding methods for assessing non-adherence (58), and GPs’ and patients’ knowledge of adherence problems (59). In our focus group study, both, patients and GPs confirmed that gp-multitool.de can be one relevant approach for overcoming these deficits in multiple dimensions.

## Data Availability

The raw data supporting the conclusions of this article will be made available by the authors, without undue reservation.
