# Supplementary material for: Integrating general practitioners’ and patients’ perspectives in the development of a digital tool supporting primary care for older patients with multimorbidity: a focus group study
Source: Front Digit Health. 2025 Jan 21;7:1499333. doi: 10.3389/fdgth.2025.1499333 (PMC11790651; doi:10.3389/fdgth.2025.1499333)
Supplement: Supplementary file 1 [file Datasheet1.pdf]

Integrating GPs' and patients' perspectives in the development of a digital tool supporting primary care for older patients with multimorbidity: a focus group study

Supplementary Material 1: Assessments included in gp-multitool.de

´

Ingmar Schäfer, Vivienne Jahns, Valentina Paucke, Tina Mallon,

Dagmar Lühmann, Martin Scherer, and Julia Nothacker

Box S1: Prioritisation of treatment goals

Therapies are intended to relieve pain or other symptoms, to promote a long life, and to maintain independence. However, not all of these therapy goals can always be taken into account equally. Please indicate how important the following treatment goals are for you in your current life situation. Each priority can only be ticked once. The decision is not always easy. So please take some time to think about it.

1) A long life is …

- ○ … most important
- ○ … priority 2
- ○ … priority 3
- ○ … priority 4
- ○ … least important

2) That I remain independent and can maintain my usual activities (e.g. shopping, or meeting other people) is …

- ○ … most important
- ○ … priority 2
- ○ … priority 3
- ○ … priority 4
- ○ … least important

3) That my medical treatments (e.g. taking medication, or visiting healthcare professionals) are limited to what is absolutely necessary is …

- ○ … most important
- ○ … priority 2
- ○ … priority 3
- ○ … priority 4
- ○ … least important

4) That I have little pain is …

- ○ … most important
- ○ … priority 2
- ○ … priority 3
- ○ … priority 4
- ○ … least important

5) That I have little other complaints (e.g. dizziness, itching, or tiredness) is …

- ○ … most important
- ○ … priority 2
- ○ … priority 3
- ○ … priority 4
- ○ … least important

Box S2: Control preferences and involvement of other healthcare professionals

1) When you think about the treatment decisions with your GP over the last twelve months: Have you been as involved as you would like?

- ○ I felt rather overwhelmed by having to make a decision
- ○ I was generally satisfied with how much I was involved
- ○ I would have liked to have been more involved

2) Since your last visit to your GP, have you consulted other healthcare professionals (i.e. people outside of your GP practice, e.g. other doctors, therapists, nurses)? If so, which ones?

____

3) Would you like other people besides you and your GP to be present when decisions about further treatment are made? If so, who?"

____

Box S3a: Activities and participation

During the last four weeks, how often were you able to organize the following areas of your life the way you wanted? It doesn't matter whether you managed this all by yourself or with support, e.g. from other people or technical devices.

1) Were you able to move freely in your home?

○ Always, or most of the time
○ Partially
○ Sometimes, or not at all
○ Not applicable or not important to me

2) Were you able to move freely outside your home?

○ Always, or most of the time
○ Partially
○ Sometimes, or not at all
○ Not applicable or not important to me

3) Were you able to do your self-care or were you cared for as you wanted? (E.g. getting dressed, washing yourself, looking after your health)

○ Always, or most of the time
○ Partially
○ Sometimes, or not at all
○ Not applicable or not important to me

4) Was your home cared for as you wanted?

○ Always, or most of the time
○ Partially
○ Sometimes, or not at all
○ Not applicable or not important to me

5) Were those who depend on you well cared for? (E.g. grandchildren, relatives in need of care, pets)

○ Always, or most of the time
○ Partially
○ Sometimes, or not at all
○ Not applicable or not important to me

Box S3b: Activities and participation (continued)

6) Were you able to eat in a way that you think is good for you?

- ○ Always, or most of the time
- ○ Partially
- ○ Sometimes, or not at all
- ○ Not applicable or not important to me

7) Were you able to do physical activities? (E.g. gardening, sports and exercises)

- ○ Always, or most of the time
- ○ Partially
- ○ Sometimes, or not at all
- ○ Not applicable or not important to me

8) Were you able to keep yourself mentally fit? (E.g. reading books or magazines, playing board games, solving crossword puzzles)

- ○ Always, or most of the time
- ○ Partially
- ○ Sometimes, or not at all
- ○ Not applicable or not important to me

9) Were you able to participate in social activities? (E.g. meeting friends, engaging politically or socially, practicing your religion)

- ○ Always, or most of the time
- ○ Partially
- ○ Sometimes, or not at all
- ○ Not applicable or not important to me

Box S4: Problems with medication

Many people use medications in their own way, which may differ from they have discussed with their GP. It would be important to know how you are using your current medications so that you and your GP can plan your further treatment well.

1) How often do you forget to take the medication?

○ (Almost) never
○ Sometimes
○ Often

2) How often do you take the medication at different times than agreed?

○ (Almost) never
○ Sometimes
○ Often

3) How often do you deliberately take more than agreed?

○ (Almost) never
○ Sometimes
○ Often

4) How often do you deliberately take less than agreed?

○ (Almost) never
○ Sometimes
○ Often

5) How satisfied are you with your medication?

○ Satisfied
○ Partially satisfied
○ Dissatisfied

6) Do you think your medication has side effects? If so, what are they?

____

Box S5a: Treatment burden

Many people have difficulty implementing measures such as regular blood sugar measurements or dietary changes at home. It would be important to find out something about your situation so that you and your GP can plan your further treatment well. Please indicate how difficult you find the following activities.

1) Visiting doctors and other health professionals

- ○ Not difficult
- ○ Partially
- ○ Very difficult
- ○ Not applicable

2) Keeping agreed appointments (e.g. travel, waiting time)

- ○ Not difficult
- ○ Partially
- ○ Very difficult
- ○ Not applicable

3) Taking your medication

- ○ Not difficult
- ○ Partially
- ○ Very difficult
- ○ Not applicable

4) Paying for medication yourself

- ○ Not difficult
- ○ Partially
- ○ Very difficult
- ○ Not applicable

5) Carrying out agreed exercises (e.g. physiotherapy)

- ○ Not difficult
- ○ Partially
- ○ Very difficult
- ○ Not applicable

6) Implementing and maintaining changes to your lifestyle (e.g. diet and physical activities)

- ○ Not difficult
- ○ Partially
- ○ Very difficult
- ○ Not applicable

Box S5b: Treatment burden (continued)

7) Monitoring your health (e.g. measuring blood sugar)

○ Not difficult
○ Partially
○ Very difficult
○ Not applicable

8) Being dependent on help from family or friends

○ Not difficult
○ Partially
○ Very difficult
○ Not applicable

9) Being dependent on a care service

○ Not difficult
○ Partially
○ Very difficult
○ Not applicable

Box S6a: Pain

1) In the last four weeks, have you had pain for several days?

- ○ Yes
- ○ No

2) In your opinion, what could be the cause of this pain?

____

3) Please indicate in the figures below where you felt this pain. If several areas of the body are affected, please report the pain that bothers you the most.

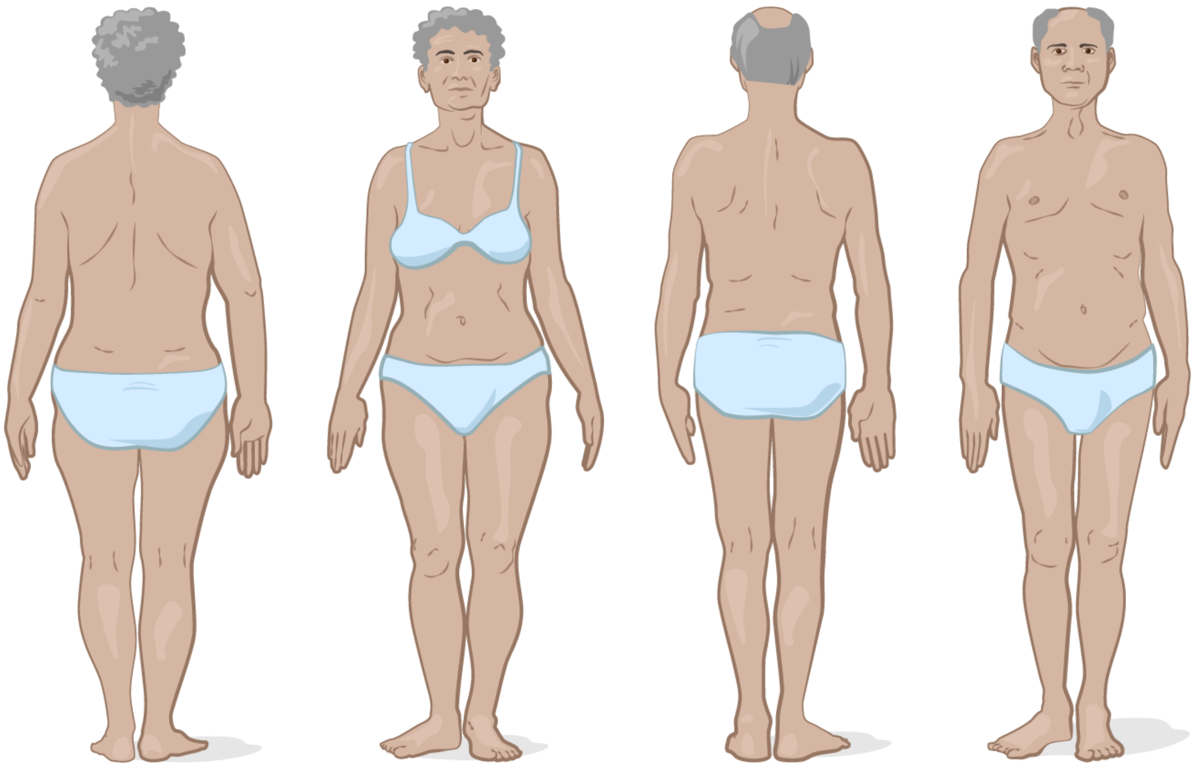

Images © Janis Vernier 2022

Box S6b: Pain (continued)

4) Please indicate in the scale below the average pain intensity during the last seven days:

no pain 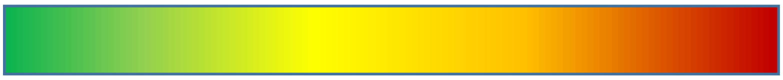 strongest pain imaginable

5) Please indicate in the scale below the greatest pain intensity during the last seven days

no pain 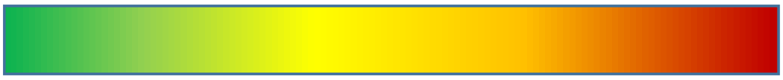 strongest pain imaginable

6) When does the pain occur?

☐ At rest
☐ During movement or under stress
☐ Other, namely: ____

How often does the pain occur?

☐ Permanent/chronic
☐ Recurring
☐ Other, namely: ____

How does the pain feel?

☐ Stabbing
☐ Dull
☐ Burning
☐ Radiating
☐ Tingling
☐ Pulling
☐ Electric
☐ Other, namely: ____

Box S7a: Other health complaints

Please indicate whether you suffer from the respective symptoms and, if so, how much you feel impaired because of them.

<u>Nervous system and memory</u>

1) Difficulty concentrating

- ○ Not applicable
- ○ Not impairing
- ○ Somewhat impairing
- ○ Strongly impairing

2) Insomnia or sleep disorders

- ○ Not applicable
- ○ Not impairing
- ○ Somewhat impairing
- ○ Strongly impairing

3) Numbness and tingling in the hands or feet

- ○ Not applicable
- ○ Not impairing
- ○ Somewhat impairing
- ○ Strongly impairing

4) Visual disturbances

- ○ Not applicable
- ○ Not impairing
- ○ Somewhat impairing
- ○ Strongly impairing

<u>Breathing and circulation</u>

5) Dizziness or circulatory problems

- ○ Not applicable
- ○ Not impairing
- ○ Somewhat impairing
- ○ Strongly impairing

6) Dyspnoea, shortness of breath (when exercising or resting) and/or tightness in the chest

- ○ Not applicable
- ○ Not impairing
- ○ Somewhat impairing
- ○ Strongly impairing

Box S7b: Other health complaints (continued)

7) Heart palpitations

- ○ Not applicable
- ○ Not impairing
- ○ Somewhat impairing
- ○ Strongly impairing

8) Cough (recurring/chronic)

- ○ Not applicable
- ○ Not impairing
- ○ Somewhat impairing
- ○ Strongly impairing

9) Nosebleeds

- ○ Not applicable
- ○ Not impairing
- ○ Somewhat impairing
- ○ Strongly impairing

<u>Digestion and urinary system</u>

10) Digestive problems

- ○ Not applicable
- ○ Not impairing
- ○ Somewhat impairing
- ○ Strongly impairing

11) Nausea

- ○ Not applicable
- ○ Not impairing
- ○ Somewhat impairing
- ○ Strongly impairing

12) Problems with the teeth, gums or dentures

- ○ Not applicable
- ○ Not impairing
- ○ Somewhat impairing
- ○ Strongly impairing

Box S7c: Other health complaints (continued)

13) Dry mouth and/or thirst

- ○ Not applicable
- ○ Not impairing
- ○ Somewhat impairing
- ○ Strongly impairing

14) Problems urinating

- ○ Not applicable
- ○ Not impairing
- ○ Somewhat impairing
- ○ Strongly impairing

15) Burning or pain when urinating

- ○ Not applicable
- ○ Not impairing
- ○ Somewhat impairing
- ○ Strongly impairing

<u>Skin</u>

16) Itching or dry skin

- ○ Not applicable
- ○ Not impairing
- ○ Somewhat impairing
- ○ Strongly impairing

17) Poor wound healing or chronic wounds

- ○ Not applicable
- ○ Not impairing
- ○ Somewhat impairing
- ○ Strongly impairing

18) Swollen legs and feet

- ○ Not applicable
- ○ Not impairing
- ○ Somewhat impairing
- ○ Strongly impairing

Box S7d: Other health complaints (continued)

Physical well-being

19) Exhaustion, tiredness, lack of energy

- ○ Not applicable
- ○ Not impairing
- ○ Somewhat impairing
- ○ Strongly impairing

20) Weakened immune system and susceptibility to infections

- ○ Not applicable
- ○ Not impairing
- ○ Somewhat impairing
- ○ Strongly impairing

21) Loss of appetite

- ○ Not applicable
- ○ Not impairing
- ○ Somewhat impairing
- ○ Strongly impairing

22) Weight loss

- ○ Not applicable
- ○ Not impairing
- ○ Somewhat impairing
- ○ Strongly impairing

23) Weight gain

- ○ Not applicable
- ○ Not impairing
- ○ Somewhat impairing
- ○ Strongly impairing

24) Night sweats

- ○ Not applicable
- ○ Not impairing
- ○ Somewhat impairing
- ○ Strongly impairing

25) Problems related to sex life

- ○ Not applicable
- ○ Not impairing
- ○ Somewhat impairing
- ○ Strongly impairing
