# Supplementary material for: Integrating general practitioners’ and patients’ perspectives in the development of a digital tool supporting primary care for older patients with multimorbidity: a focus group study
Source: Front Digit Health. 2025 Jan 21;7:1499333. doi: 10.3389/fdgth.2025.1499333 (PMC11790651; doi:10.3389/fdgth.2025.1499333)
Supplement: Supplementary file 2 [file Datasheet2.pdf]

Integrating GPs' and patients' perspectives in the development of a digital tool supporting primary care for older patients with multimorbidity: a focus group study

Supplementary Material 2: Exemplary quotes of study participants

Ingmar Schäfer, Vivienne Jahns, Valentina Paucke, Tina Mallon,

Dagmar Lühmann, Martin Scherer, and Julia Nothacker

Table S1a: Exemplary quotes of study participants

GP perspective	Patient perspective
Prioritisation of treatment goals	
“I can imagine that some people also say: ‘For God's sake, if I [choose ‘not so important to me’ for the treatment goal “a long life’], then I won't get [my] tablets any more’.” (28 October 2022, man, 58 years old, practicing for 28 years).<br>*‘‘To live long’, for example, is a very neutral statement, but ‘to prolong life’, [...] reminds me of advance directives. Then [...] [most people] imply ‘to prolong suffering’.” (28 October 2022, woman, 55 years old, practicing for 29 years).*	*“I said I want a long life because I know my wife will have it. So I want to be with her for a long time. But whether it's a big priority for me [...] is questionable. It's not all that easy, the chains of thought you have to go through.” (22 March 2023, man, 68 years old, tertiary education).*
Control preferences and involvement of other healthcare professionals	
“You have to discuss this at some point anyway [...] – Does [the patient] leave it all to you or [...] does he want to do the whole thing self-determined?” (20 January 2023, woman, 61 years old, practicing for 36 years).<br>*“I cannot imagine most patients over the age of 80 thinking so abstractly about how a doctor-patient relationship and decision-making should take place.” (9 December 2022, woman, 55 years old, practicing since 29 years).*<br>*“I would never accept that [I] should make all decisions myself that affect [a patient] medically. [...] In fact, many patients come with this request. Then they say: ‘Yes, doctor, you are the expert. You should decide’. But that's exactly what [...] I then reject in conversation and say: ‘No, you're the expert. You are the one who has to decide for yourself’.” (9 December 2022, man, 54 years old, practicing for 24 years).*	*“‘Being overwhelmed’ naturally has a slightly negative flavour. So when it says: ‘I'm quite happy that I don't have to help make decisions’, then perhaps that's a bit more positive. So, ‘I feel overwhelmed‘. Who likes to tick that box?“ (22 March 2023, man, 68 years old, tertiary education).*<br>*“Actually, you feel overwhelmed. You're not a doctor. You can only say that if there are two options: ‘one option seems better to me’. [...]. Basically, I go [to the doctor] because I can't decide, because I'm not competent. And you want help.” (22 March 2023, woman, 74 years old, secondary education).*
Social contacts	
“Ultimately, this is about different things, on the one hand it's about loneliness and social structures and on the other [...] about things that can regularly help [patients] at home. [...] That's why I wouldn't necessarily find the total score here [...] so great [...], but rather the individual questions would be more important to me.” (20 January 2023, woman, 26 years old, practicing for 2 years).<br>*“I somehow don't quite agree with the wording of the introduction [...]. ‘Many older people have little contact with other people, some of them like to be alone’. [...] I find that [...] almost a bit discriminatory.” (11 January 2023, man, 57 years old, practicing for 30 years).*	

GP: general practitioner

Table S1b: Exemplary quotes of study participants (continued)

GP perspective	Patient perspective
Activities and participation	
"Well, I just think that someone who goes to work and does sport [...] doesn't want to answer a question about whether he can move around freely in his home. So then he would [...] perhaps simply lose the desire to fill it out [the assessment] seriously and then I can't really do much with the information afterwards." (20 January 2023, woman, 54 years old, practicing for 28 years).	*"What I've generally noticed is: sport in general and fitness. It's in here with the cardiac sports group, but [...] if someone [...] still goes to the gym or something like that. We don't find that here at all." (27 April 2023, man, 73 years old, primary education).*
"Of course, you also have to be careful. It's very invasive of the patient's privacy and therefore, as a patient, I would be completely irritated at a certain point: 'So, what do they want from me here?'" (28 October 2022, woman, 55 years old, practicing for 29 years).	
Problems with medication	
"I think the approach is very generic. I would actually make it more personal. [...] 'I have prescribed medication for you and I know that taking it is not always easy and would like to ask you [...] whether it works regularly or not so often'." (20 January 2023, woman, 61 years old, practicing for 36 years).	*"So, if I take water tablets, I have completely different symptoms than if I take antihypertensives [...]. And so you might have to make a more differentiated list of the medication I'm taking. […] 'You're taking water tablets. Did it work, did it not work? What complications have occurred?'" (19 April 2023, man, 69 years old, primary education).*
Treatment burden	
"You know, [...] the health insurance companies don't authorise the transports. [...] And we [have to] keep saying no [...] to the patient, even though I would personally organise everything. [...] You have to be careful [...] that you don't run out of the practice screaming and at some point think: 'What is this, it can't be right'." (28 October 2022, woman, 55 years old, practicing for 29 years).	
"For example, the question of being dependent on family and friends. Today I had an elderly lady who said: 'What do you think? When do I have to go into a nursing home? I think I'm a burden to my daughter. We live next door to each other.' [...] I would never have thought that she would need so much care or help from her daughter. Because she is still very fit and mentally clear." (20 January 2023, woman, 54 years old, practicing for 28 years).	
"Why does everything have to be labelled 'difficult'? [...] Maybe it is someone who has amazing children or lives with his children and they have the opportunity to drive him to all the doctors and get it done. Then you can write 'unproblematic'. [...] There are people for whom this is not difficult." (28 October 2022, man, 58 years old, practicing for 28 years).	

GP: general practitioner

Table S1c: Exemplary quotes of study participants (continued)

GP perspective	Patient perspective
Pain	
“So for an […] assessment [that] is supposed to cover a longer period of time, I would consider the current pain intensity to be less important than the average pain intensity. […] I actually think [the highest pain intensity] is the most important, it integrates the pain over a certain period of time and then actually gives the best information regarding prioritisation, where the pain is the most severe.” (9 December 2022, man, 54 years old, practicing for 24 years).<br>*“If we try […] to deal with it in a generally non-discriminatory way, then gender simply makes no difference here. If we now make it so schematized that we cannot differentiate between […] cultures or skin colour […] then I don’t think anything is lost. So the question is whether we should not take so much consideration […] from the start and simply proceed in a schematized way from the start.” (9 December 2022, man, 54 years old, practicing for 24 years).*	*"If there is pain that really means danger, then I would be happy if it was colour-coded. But if it's just severe pain, why do I have to [choose something] red? The number 10 counts, that's the highest [...] degree of pain. That's enough, I just don't need any colour. Only if [...] it says, ‘Man, you have to call [the rescue service]’.” (15 June 2024, man, 81 years old, secondary education).*<br>*“[I] think it's important that a question is asked, [...] where the pain comes from or [...] what caused the pain. [...] I sometimes have joint pain, which can be a side effect of tablets. It could also be [...] that you made an unusual movement somewhere.” (19 April 2023, woman, 74 years old, tertiary education).*
Other health complaints	
“What I like about this questionnaire is that the question is not ‘Do you have it or not?’ but ‘How much does it affect you?’ Because that immediately gives a certain weighting. So it's definitely the case that someone has or perceives fatigue, tiredness or lack of energy, but that doesn't bother them because they assume that they are getting older and that this is completely normal.” (9 December 2022, man, 54 years old, practicing for 24 years).<br>*“I would have put the sensory organs in one group, so I wouldn't have put 'visual disorders' under the 'nervous system'. I would have liked to have included 'hearing disorders' too, just in case, and I wouldn't have necessarily put 'nosebleeds' under 'breathing and circulation' either. Not everyone who has high blood pressure has nosebleeds and conversely not everyone who has nosebleeds has high blood pressure.” (11 January 2023, woman, 57 years old, practicing for 31 years).*	*"But if he gets [this information], maybe he would get a different picture or [make] a different diagnosis. I think it's important." (27 April 2024, woman, 81 years old, secondary education)*
Medication reviews	
“I think I would leave out the word 'detailed' at the end [of the invitation letter] so as not to raise false expectations. [...] I think some of my patients then plan to have an hour of coffee with me. [...] I [would] even add [...] 'we are planning 20 minutes for the appointment', [...] so that I can limit it from the start, but then maybe build on it in the next appointment.” (7 June 2023, woman, 61 years old, practicing for 31 years).	

GP: general practitioner

Table S1d: Exemplary quotes of study participants (continued)

GP perspective	Patient perspective
Management of assessments, patients and users	
"It's rather a personal impression. Well, I can relate to that nonetheless. Sometimes you are surprised. My 87-year-old mother would probably be able to use it. My 84-year-old father too." (7 June 2023, man, 57 years old, practicing for 30 years).	*"Well, that also bothered me with the [pre-filled radio buttons when prioritising treatment goals]. That there was always something predefined at the front. The selection was fine for me. But I thought to myself, 'why is the point [predefined] already?'" (15 June 2023, woman, 66 years old, secondary education).*
"I don't yet have a clear idea of how the patients for whom it is relevant will get access to it. Well, it's obviously really nice to have a hyperlink and fill out [the assessment] at home. And then we look at it together or I look at it myself and pick out individual points. But most patients [...] for whom it is very relevant are over 80 and can't do much with hyperlinks. It is of course a question of time, but at the moment that is certainly still the case." (7 June 2023, woman, 52 years old, practicing for 26 years).	*"The doctor also types it into his computer. When I sit there and tell him how I'm doing or what problems I'm having, he writes it down so that he knows next time. And whether I've already answered the questionnaire [in a digital form makes no difference to me]." (5 June 2023, woman, 79 years old, primary education).*
"What I could also imagine: We also have children [of patients] who are around 60 or so and who can then perhaps report on the pain of their parents [...] – in the presence of the parents or by asking the parents [...] or simply by observing them. One lady always has back and hip problems, but can no longer express herself. But the children can see how she is doing or that transport is no longer possible or something like that. And then the daughter could fill it out. And then she comes to my consultation and then we discuss how we can change the medication [...] – without me having to make a home visit." (31 May 2023, woman, 54 years old, practicing for 28 years).	
Literature search and CPG data base	
"In terms of user friendliness, it looks very simple and logical to me. I think that's very good. In the practice, in everyday life there's often no time. And these are also things that I personally research in the evening on certain topics that interest me or that I want to know. I think it is a great feature." (7 June 2023, woman, 53 years old, practicing for 16 years).	
"I probably wouldn't use [the literature search], but simply because I know how to use PubMed and I feel comfortable with it. If someone isn't familiar with PubMed, it could of course be that this is a good additional tool." (7 June 2023, woman, 36 years old, practicing for 8 years).	
"The DEGAM guidelines have the advantage of always having a brief overview. And if I do, then I actually only have time to look at the brief overview and not directly via the AWMF [which is offered by gp-multitool.de]. So I have the impression that I would not use a tool like this." (7 June 2023, man, 57 years old, practicing for 30 years).	

GP: general practitioner
